# THE EFFECT OF AN ARTIST-IN-RESIDENCE PROGRAM ON SELF-REPORTED LONELINESS IN OLDER ADULTS

**DOI:** 10.1093/geroni/igz038.208

**Published:** 2019-11-08

**Authors:** catherine Richmond-Cullen

**Affiliations:** the university of Scranton, Scranton, Pennsylvania, United States

## Abstract

The study, funded by the Pennsylvania Council on the Arts and the Pennsylvania Department of Aging, measured the effect that an artist in residence program (conducted by state-vetted professional teaching artists) had on self-reported loneliness in older adult. All participants were aged sixty years or older and participated in programming in state-funded adult community centers located in fourteen sites throughout the Commonwealth of Pennsylvania. Artists offered 10 sessions in creating and critiquing art to older citizens in the artists’ respective art forms including performing arts, visual arts and multidisciplinary/interdisciplinary arts. Through pre and post-tests, changes in loneliness were measured using the Revised UCLA Loneliness Scale. The data revealed that there was a significant correlation between a self-reported decrease in feelings of loneliness and participation in a program conducted by professional artists. . It was proposed that findings from the study could influence the quality of programs provided by state-funded adult community centers in Pennsylvania and increase funding levels to adult community centers throughout the Commonwealth of Pennsylvania.

